# Pesticide Acute Toxicity Is a Better Correlate of U.S. Grassland Bird Declines than Agricultural Intensification

**DOI:** 10.1371/journal.pone.0057457

**Published:** 2013-02-20

**Authors:** Pierre Mineau, Mélanie Whiteside

**Affiliations:** 1 Science and Technology Branch, Environment Canada, National Wildlife Research Centre, Carleton University Campus, Ottawa, Canada; 2 Pest Management Regulatory Agency, Health Canada, Ottawa, Canada; Monash University, Australia

## Abstract

Common agricultural birds are in decline, both in Europe and in North America. Evidence from Europe suggests that agricultural intensification and, for some species, the indirect effects of pesticides mediated through a loss of insect food resource is in part responsible. On a state-by-state basis for the conterminous Unites States (U.S.), we looked at several agronomic variables to predict the number of grassland species increasing or declining according to breeding bird surveys conducted between 1980 and 2003. Best predictors of species declines were the lethal risk from insecticide use modeled from pesticide impact studies, followed by the loss of cropped pasture. Loss of permanent pasture or simple measures of agricultural intensification such as the proportion of land under crop or the proportion of farmland treated with herbicides did not explain bird declines as well. Because the proportion of farmland treated with insecticides, and more particularly the lethal risk to birds from the use of current insecticides feature so prominently in the best models, this suggests that, in the U.S. at least, pesticide toxicity to birds should be considered as an important factor in grassland bird declines.

## Introduction

Many grassland (the North American favoured term) or farmland (the European favoured term) bird species have undergone range contractions and/or population declines in recent decades in both northern Europe and North America. Indeed, North American analyses indicate that grassland birds as a group are declining faster than birds from other biomes [1], [2]. Rodenhouse and colleagues [3] reported that 215 species of neotropical migrants use agricultural areas in North America. Several neotropical migrant species have, over the years, been listed as threatened or endangered or are candidates for listing, and agriculture is implicated in the decline of many. Most investigators have blamed farmland bird declines on some aspect of agricultural intensification: the shift to larger fields, row and field crop monocultures and denser more uniform crop structure, loss of native pasture and other semi-natural habitats, increase in autumn sowings, increased inputs of fertilizers and pesticides to name a few [4]–[10]. Increased predation as a result of habitat change has also been invoked [11]. As one component of agricultural intensification, herbicides and insecticides have been linked to population declines in some bird species in the UK, primarily via indirect, food-mediated effects [5], [12]. Fox [13] reported that farmland birds in Denmark had not declined as they had in the UK and that a notable difference in crop intensification with the latter had been a clear and gradual reduction in agrochemical inputs. More recently, Geiger and colleagues [14] in a large study with study sites in 8 European countries concluded that: “*Out of the 13 studied components of agricultural intensification, use of pesticides, especially insecticides and fungicides, had the most consistent negative effects on the species diversity of plants, carabids and ground-nesting farmland birds, and on the potential for biological pest control*.” Bird diversity specifically was most highly correlated with fungicide use although the authors pointed out that fungicide use and insecticide use were highly correlated.

Historically, insecticide use in North America has been very different from that in the UK – or Denmark. Specifically, large quantities of products of very high toxicity to birds have been used for decades despite evidence that poisonings were frequent even when products were applied according to label direction [15]–[17]. The Avian Incident Monitoring System (AIMS), a joint project of the U.S. Environmental Protection Agency (EPA) and the American Bird Conservancy (ABC), listed 113 pesticides which have caused direct bird mortality [18]. An analysis of a large number of avian field studies [19], [20] suggests that avian kills were a normal corollary of insecticide use in many crops grown in North America. For example, analyses of granular insecticide use patterns in western Canada indicated that the abundance of several common species was negatively correlated with these toxic insecticides [21]. Pimentel [22], in an oft-cited study, estimated that pesticide-induced direct mortality numbered approximately 67 million per year in the U.S. He based this estimate on the fact that 160 million ha of cropland received a very heavy dose of pesticides per year (3 kg a.i./ha on average – including a number of very toxic pesticides), a breeding density of 4.2 birds per ha (from census plot data) and a conservative kill estimate of 10% of exposed birds. This estimate ignored kills of wintering or migrant birds which could be substantial [17]. This estimate was undoubtedly quite conservative. Mineau [23] estimated, based on several industry-led field studies that at its peak, a single use pattern (for corn rootworm) of a single insecticide (carbofuran), in a single crop (corn) was killing 17 to 91 million songbirds annually in the U.S. Midwest. This raises the question as to whether broader patterns of grassland bird species declines can be, in part at least, explained by the direct toxic effects of pesticides.

An analysis of pesticide use patterns in the US does suggest that the situation is improving as a result of the gradual withdrawal of the most toxic products, largely because of human health concerns [17]. The current analysis considered bird trends from 1980 to 2003; there is evidence that the acute lethal risk to birds was already dropping during the second half of that period.

## Methods

### Pesticide use data

We assembled State by State pesticide use data from a number of sources. The United States Department of Agriculture (USDA) National Center for Agricultural Statistics (NASS) provides crop acreage, one time pesticide application rate, and number of applications, but these data are for major crops only and are only provided for certain states. A complete matrix of State*crop*active ingredient combinations was obtained from the National Center for Food and Agriculture Policy (NCFAP). For the years circa 1992 (1990–1993) and circa 1997 (1994–1998) NCFAP reported crop acreage, the percentage of crop treated with a given active ingredient, and the cumulative yearly application rate to each treated acre based on NASS data as well as a plethora of State surveys, personal communications with extension specialists etc… . Unfortunately, one-time application rates and the annual number of repeat applications were not reported by NCFAP and thus had to be retrieved or calculated from a number of sources including the original sources cited by NCFAP where possible. The full methodology is detailed in Mineau and Whiteside [17]. For the current analysis, only the1992 data were used as they fell in the midpoint of the period for which we compiled bird trend estimates and before a number of restrictions on toxic organophosphorous (OP) insecticide use.

### Bird trend data

We used data from the United States Geological Survey (USGS) Breeding Bird Survey (BBS) to calculate species trends. The BBS is an annual roadside count of standardized 39.4 km routes with stops at 0.8 km interval. Volunteer observers record all species seen or heard within a 0.4 km radius over a 3 min. census period. For the whole country analysis, we relied on the bird trend information provided for the ‘grassland’ bird species guild as generated by the USGS Migratory Bird Division for each State. We used the trends reported for route regression analyses run between 1980 and 2003 (http://www.mbr-pwrc.usgs.gov/bbs/trend/guild03.html). For any given State, a trend analysis was provided only if the species was recorded on 14 routes or more. Thus, the number of species showing positive or negative trends was taken to be a self-equilibrating index of the health of grassland birds across the conterminous U.S. given that different grassland species dropped in and out of the analysis in each State dependent on their range and abundance (Table 1). We considered restricting the analysis to only those species showing statistically significant trends but this would have greatly reduced sample size, especially the number of statistically-significant increases, making our use of logistic modeling difficult.

**Table 1:** Grassland species ordered by the number of total state*species declines and the number of recorded declining or increasing trends (1980–2003) for the 45 conterminous American States.

SPECIES		Total declines(significant declines)	Total increases (significant increases)	Recorded killed in pesticide field trials
Ammodramus savannarum	Grasshopper sparrow	25 (15)	3 (1)	X
Pooecetes gramineus	Vesper sparrow	18 (8)	3 (0)	X
Phasianus colchicus	Ring-necked pheasant	19 (10)	6 (2)	X
Eremophila alpestris	Horned lark	24 (16)	5 (2)	X
Passerculus sandwichensis	Savannah sparrow	12 (5)	8 (3)	X
Circus cyaneus	Northern harrier	8 (2)	8 (1)	X
Bartramia longicauda	Upland sandpiper	8 (2)	4 (1)	
Sturnella neglecta	Western meadowlark	17 (11)	4 (1)	X
Dolichonyx oryzivorus	Bobolink	12 (7)	5 (1)	X
Spiza americana	Dickcissel	8 (3)	10 (2)	X
Numenius americanus	Long-billed curlew	4 (0)	3 (1)	
Sturnella magna	Eastern meadowlark	33 (30)	0 (0)	X
Calamospiza melanocorys	Lark bunting	6 (3)	1 (1)	
Athene cunicularia	Burrowing owl	2 (0)	3 (1)	X
Cistothorus platensis	Sedge wren	2 (0)	3 (1)	
Tympanuchus phasianellus	Sharp-tailed grouse	2 (0)	1 (0)	
Ammodramus bairdii	Baird’s sparrow	2 (1)	0 (0)	
Calcarius ornatus	Chestnut-collared longspur	3 (2)	0 (0)	X
Buteo regalis	Ferruginous hawk	1 (0)	4 (0)	
Tympanuchus cupido	Greater prairie chicken	1 (0)	0 (0)	
Ammodramus leconteii	Le Conte’s sparrow	1 (0)	1 (1)	X
Asio flammeus	Short-eared owl	2 (1)	1 (0)	X
Anthus spragueii	Sprague’s pipit	1 (0)	1 (0)	X
Aimophila cassinii	Cassin’s sparrow	4 (4)	0 (0)	X
Ammodramus henslowii	Henslow’s sparrow	2 (2)	0 (0)	
Charadrius montanus	Mountain plover	0 (0)	1 (1)	

Also indicated is whether the species has ever been recorded killed during pesticide field trials based on [19] augmented by similar studies on granular insecticides.

### Agricultural variables

#### Agricultural Intensity and change:

We developed both ‘static’ and ‘change’ measures of agricultural intensity. Our static indicator of State agricultural intensity was developed to be analogous to the pesticide indicators. It is defined here as the percentage of total agricultural land that is allocated to active cropping. Using the NASS categories of land use, agriculture intensity was obtained by dividing the amount of ‘cropland for crops’ by the amount of total farmland including ‘cropland for crops’ which in turn included ‘fallow’, ‘pasture’, and ‘grazed forest’.

The change indicators relevant to grassland birds were deemed to be changes in the land area devoted to permanent and cropped pasture. These were calculated simply as the 2002 pasture or crop pasture areas minus the 1978 pasture or crop area pastures expressed as a proportion of the 1978 area for each State.

#### Herbicide use:

Very few crops in North America are grown without the use of herbicides. Therefore, the acreage of crops to which herbicides have been applied divided by the area of ‘total farmland’ is another measure of agricultural intensification. From the pesticide dataset described earlier, areas treated were summed for all herbicide*crop combinations. Because most crops are treated with multiple herbicides, the total acreage treated with herbicides often exceeded the ‘total farmland’ value which resulted in a value greater than 1.0 as shown in Table 2. If that was the case, the total percentage was capped at 1.0 or 100% prior to analysis.

**Table 2:** Agricultural variables and BBS summarised results for each State.

State	Agricultural intensity	Proportional change in permanent pasture (1978–2002)	Proportional change in crop pasture (1978–2002)	Lethal pesticide risk	Insecticide use	Herbicide use	No. increasing species (no. significant increases)	No. declining species (no. significant declines)
ALABAMA	0.34	–0.02	–0.52	0.25	0.53	1.01	1 (1)	2 (1)
ARIZONA	0.03	–0.02	0.33	0.02	0.09	0.08	0 (0)	2 (1)
ARKANSAS	0.59	0.13	–0.42	0.06	0.22	1.23	1 (1)	1 (1)
CALIFORNIA	0.29	–0.05	–0.21	0.14	0.54	0.4	2 (1)	6 (2)
COLORADO	0.25	–0.02	0.41	0.02	0.04	0.29	5 (0)	8 (5)
CONNECTICUT	0.62	–0.36	–1.39	0.08	0.22	0.64	0 (0)	1 (1)
FLORIDA	0.26	–0.14	–0.17	0.18	0.35	0.58	0 (0)	1 (1)
GEORGIA	0.56	–0.07	–0.66	0.38	0.74	1.39	0 (0)	2 (2)
IDAHO	0.37	0.00	0.00	0.04	0.06	0.3	1 (0)	6 (3)
ILLINOIS	0.91	0.26	–1.83	0.09	0.14	1.96	1 (0)	9 (7)
INDIANA	0.83	–0.01	–1.43	0.09	0.13	1.97	1 (0)	7 (6)
KANSAS	0.56	–0.06	–0.27	0.06	0.12	0.61	2 (1)	8 (6)
KENTUCKY	0.46	0.77	–0.59	0.04	0.08	0.62	2 (0)	2 (1)
LOUISIANA	0.59	–0.08	–0.30	0.29	0.75	1.99	0 (0)	2 (1)
MAINE	0.64	–0.64	–1.04	0.1	0.21	0.35	1 (0)	2 (1)
MARYLAND	0.82	0.67	–1.26	0.06	0.25	1.34	1 (1)	4 (3)
MASSACHUSETTS	0.6	–0.28	–1.57	0.12	0.29	0.5	0 (0)	3 (1)
MICHIGAN	0.83	0.29	–0.84	0.12	0.21	1.34	1 (0)	9 (4)
MINNESOTA	0.84	0.15	–1.21	0.05	0.09	1.56	1 (0)	12 (3)
MISSISSIPPI	0.56	0.20	–1.09	0.54	0.97	1.91	0 (0)	2 (1)
MISSOURI	0.49	0.10	–0.65	0.03	0.08	0.82	2 (1)	6 (4)
MONTANA	0.24	–0.05	0.31	0.01	0.02	0.19	8 (1)	9 (3)
NEBRASKA	0.45	–0.01	–0.24	0.08	0.14	0.64	3 (0)	9 (1)
NEVADA	0.05	0.01	0.39	0	0.01	0.01	2 (1)	0 (0)
NEW HAMPSHIRE	0.64	0.47	–1.30	0.03	0.15	0.28	0 (0)	2 (2)
NEW JERSEY	0.73	–0.23	–0.46	0.11	0.26	1.24	0 (0)	2 (2)
NEW MEXICO	0.03	0.01	0.43	0	0.01	0.08	1 (0)	6 (5)
NEW YORK	0.59	–0.03	–1.34	0.09	0.18	0.65	0 (0)	9 (5)
NORTH CAROLINA	0.67	0.03	–0.48	0.29	0.56	1.5	0 (0)	2 (1)
NORTH DAKOTA	0.64	0.06	–0.20	0.02	0.03	0.87	11 (6)	8 (4)
OHIO	0.8	–0.20	–0.79	0.07	0.1	1.51	2 (1)	7 (6)
OKLAHOMA	0.32	0.00	0.19	0.05	0.11	0.32	1 (0)	6 (3)
OREGON	0.22	0.01	0.19	0.04	0.08	0.54	2 (1)	5 (4)
PENNSYLVANIA	0.7	0.07	–0.73	0.07	0.25	0.84	2 (0)	5 (4)
SOUTH CAROLINA	0.59	–0.19	–0.49	0.14	0.44	1.46	0 (0)	1 (0)
SOUTH DAKOTA	0.35	–0.07	0.07	0.04	0.05	0.53	4 (1)	9 (4)
TENNESSEE	0.46	–0.15	–0.41	0.1	0.26	0.8	2 (0)	1 (1)
TEXAS	0.17	0.05	0.09	0.03	0.08	0.21	1 (0)	7 (5)
UTAH	0.13	0.04	0.18	0.01	0.01	0.09	3 (0)	6 (1)
VERMONT	0.49	0.11	–2.02	0.01	0.03	0.36	1 (0)	2 (2)
VIRGINIA	0.42	–0.12	–0.36	0.09	0.18	0.69	0 (0)	3 (2)
WASHINGTON	0.44	0.12	–0.23	0.06	0.17	0.82	2 (0)	6 (2)
WEST VIRGINIA	0.28	–0.22	–0.66	0.01	0.04	0.12	0 (0)	2 (2)
WISCONSIN	0.65	0.05	–0.93	0.12	0.18	0.89	2 (1)	11 (8)
WYOMING	0.06	–0.03	0.48	0	0	0.04	6 (1)	4 (0)

#### Insecticide use:

This variable was calculated in the same way as the herbicide use variable. There is less insecticide than herbicide use and the proportion of farmland treated never exceeded 1.0 or 100%.

#### Lethal pesticide risk:

This variable is the estimated lethal risk from the use of the insecticides. It was based on the logistic models developed from results of avian field studies [17], [19].

The process can be summarized as follows: As a first step, a measure of acute pesticide toxicity for birds ranging from 20 to 1,000 grams (a weight range that covers most bird species found dead in farm fields and which corresponds also to the range of generic body weights considered in U.S. and Canadian risk assessments for pesticide registration) is obtained by applying species sensitivity distribution techniques [24]. A probability that birds will be killed by a given pesticide application is then derived from a model that uses logistic multiple regression with the finding of bird carcasses in a large sample of field studies as the endpoint of interest. Note that this index does not incorporate non lethal toxic effects on birds, or indirect effects such as loss of habitat or food source from pesticide use. Aside from the toxicity of the pesticide active ingredient, the model makes use of the application rate and physico-chemical properties of pesticides. Validation of the model for a sample of studies in field crops indicated that more than 81% of studies were correctly classified – as to whether they gave rise to mortality or not [17].

One recognized weakness of the approach is that the empirical models relating mortality to pesticide toxicity and to the other independent variables were derived entirely from foliar applications of pesticides. Because formulation-specific data (i.e. whether the material was applied as a spray or a granular formulation) were not available for many products, this problem was ignored in the current analysis.

Only insecticides were considered for the acute toxicity variable. Realistically, acute avian risk from herbicide or fungicide use is minimal. The models were run using the average application rates for each crop*pesticide*State combination (8491 unique combinations). The overall risk, expressed as the proportion of the farmland area over which avian mortality was likely, was cumulated by State. Mineau and Whiteside [17] also obtained a data set of individual insecticide applications from California (the only State where individual applications are entered into a database) and were able to ascertain that the predicted risk from a given pesticide cumulated for each separate application was similar to the risk calculated from the State application rate average.

### Statistical treatment

Table 1 provides a summary of the species included in the analysis, the number of states in which trend analysis was attempted as well as the number of positive and negative trends (both significant and not) for those States having adequate coverage. The six independent indices (agricultural intensity, proportional change in permanent and cropped pasture, herbicide use, insecticide use and lethal pesticide risk) as well as the number of positive and negative bird trends for the grassland bird guild are given for each State in Table 2. The four static predictors (all proportions) were submitted to arcsin transformation; the proportional changes were left in their native state. A correlation matrix was run for all independent variables (Table 3).

**Table 3:** Correlation matrix of predictor variables. Significant (P< 0.05) correlation coefficients are shown in bold.

	Farming Intensity	Lethal pesticide risk	Insecticide Use	Herbicide Use	Change in permanent pasture	Change in cropped pasture
Farming Intensity	1.00					
Lethal pesticide risk	**0.42**	1.00				
Insecticide Use	**0.30**	**0.94**	1.00			
Herbicide Use	**0.87**	**0.59**	**0.47**	1.00		
Change in permanent pasture	**0.27**	–0.03	0.05	**0.25**	1.00	
Change in cropped pasture	–**0.80**	–**0.42**	–**0.33**	–**0.64**	–**0.30**	1.00

In the regression analysis, each state*bird combination represents a data point. This means that States with more reported trends (with more species surveyed on more than 14 routes) are weighted more heavily in the analysis. Modeling was carried out with the GLZ module of STATISTICA (Version 10) with a logit regression model. Model strength was assessed with Akaike Information Criterion (AIC) values and Delta AIC values which are simple increases in AIC from the best model. The relative strength of the predictor variables was assessed with summed Akaike weights [25].

## Results

The correlation matrix shows that most of the predictor variables were inter-correlated (Table 3). Correlations were particularly high between insecticide use and acute risk (r = 0.94) and between intensification and the change in cropped pasture (r = 0.80). The performance of all possible logit regression models is summarised in Table 4. Models are ordered by increasing delta AIC values. The proposed rule of thumb is that models with Delta AIC values of less than 2 are equally plausible [25]. Summed Akaike weights for the 6 predictor variables are provided in Table 5. On that basis, it might be tempting to conclude that ‘change in cropped pasture’ was the most plausible predictor followed by lethal insecticide use. However, the very high correlation between ‘lethal pesticide risk’ and ‘insecticide use’ (with the third highest summed Akaike weight) suggests that the importance of insecticides has been underestimated because of the partition of Akaike weights between those two variables. Because of the strong inter-dependencies of the variables (the correlation between ‘change in cropped pasture’ and ‘farming intensity’ might similarly cloud our assessment of the relative strength of the predictors), we repeated the analysis with only ‘change in cropped pasture’, ‘change in permanent pasture’ and ‘lethal pesticide risk’ as predictor variables. Summed Akaike weights were 0.23 for ‘lethal pesticide risk’, 0.17 for ‘change in cropped pasture’ and 0.07 for ‘change in permanent pasture’.

**Table 4:** Summary of model results for the logit regression and 5 predictor variables.

Change in cropped pasture	Change in permanent pasture	Farming Intensity	Lethal pesticide risk	Insecticide Use	Herbicide Use	d.f.	Delta AIC	wi (Akaike weights)
1			1		1	3	0.00	0.101
1		1	1			3	0.05	0.099
1			1			2	0.70	0.071
1				1		2	1.65	0.044
			1			1	1.71	0.043
1		1	1	1		4	1.76	0.042
1		1	1		1	4	1.77	0.042
1			1	1	1	4	1.80	0.041
1	1	1	1			4	1.91	0.039
1	1		1		1	4	1.96	0.038
1		1		1		3	1.97	0.038
1				1	1	3	2.10	0.035
1	1		1			3	2.49	0.029
1			1	1		3	2.70	0.026
1	1			1		3	3.21	0.020
1		1	1	1	1	5	3.48	0.018
1	1	1		1		4	3.56	0.017
			1		1	2	3.62	0.017
			1	1		2	3.65	0.016
		1	1			2	3.66	0.016
	1		1			2	3.68	0.016
1	1	1	1		1	5	3.69	0.016
1	1	1	1	1		5	3.70	0.016
1	1		1	1	1	5	3.79	0.015
1	1			1	1	4	3.82	0.015
1		1		1	1	4	3.88	0.015
				1		1	4.47	0.011
1	1		1	1		4	4.49	0.011
1						1	4.49	0.011
		1	1		1	3	4.57	0.010
1		1				2	5.06	0.008
1	1	1	1	1	1	6	5.46	0.007
			1	1	1	3	5.51	0.006
	1		1		1	3	5.52	0.006
1	1	1		1	1	5	5.52	0.006
	1		1	1		3	5.60	0.006
		1		1		2	5.60	0.006
		1	1	1		3	5.62	0.006
	1	1	1			3	5.65	0.006
1	1					2	5.66	0.006
				1	1	2	6.22	0.005
1	1	1				3	6.23	0.004
1					1	2	6.26	0.004
	1	1	1		1	4	6.47	0.004
	1			1		2	6.47	0.004
		1	1	1	1	4	6.51	0.004
1		1			1	3	6.60	0.004
		1		1	1	3	6.96	0.003
	1		1	1	1	4	7.33	0.003
1	1				1	3	7.48	0.002
	1	1		1		3	7.55	0.002
	1	1	1	1		4	7.59	0.002
1	1	1			1	4	7.64	0.002
	1			1	1	3	8.21	0.002
	1	1	1	1	1	5	8.35	0.002
	1	1		1	1	4	8.95	0.001
		1				1	12.59	0.000
					1	1	12.98	0.000
	1	1				2	14.37	0.000
		1			1	2	14.40	0.000
	1				1	2	14.68	0.000
	1	1			1	3	16.12	0.000
	1					1	19.89	0.000

Delta AICs and Akaike weights are provided for each model.

**Table 5:** Summed Akaike weights for all predictor variables and logit models given in table 4.

Variable	Akaike weights
Change in cropped pasture	0.844
Lethal pesticide risk	0.775
Insecticide use	0.446
Farming intensity	0.436
Herbicide use	0.425
Change in permanent pasture	0.299

Indeed, even in the full list of models with all 5 predictors, ‘lethal pesticide risk’ offered the best single-predictor model. The second best single-predictor model was with ‘insectide use’ and ‘change in cropped pasture’ came in third place. ‘Lethal pesticide risk’ as a predictor variable was 3.9 times more plausible than ‘change in cropped pasture’ based on the evidence ratio (Table 4).

## Discussion

As reviewed by Murphy [9] – See citations therein), few North American studies have managed to link specific agricultural practices with bird population trends, unlike the situation that prevails in the UK and, to a lesser extent, other European countries. Murphy [9] attempted to look for correlations between changes in certain agricultural habitat types (e.g. pasture, cropland or rangeland) with BBS trends for grassland and shrub species in the Eastern and central U.S. He found that none of the variables examined exhibited a clear negative relationship with population trends. On the other hand, positive population trends were associated with increases in rangeland, but also with increases in harvested cropland. Only when all species (whether grassland or shrub species) were combined was he able to obtain negative correlations of population trend with specific agricultural habitat types – e.g. cover crops.

When regressed one by one, all variables examined in our analysis had the expected effect on bird trends except for loss of permanent pasture. An increase in permanent pasture was associated with no trend or even a very slight trend to more declining species. It was the reverse for changes in the area under cropped pasture; i.e. increase in the area of cropped pasture equals more bird increases. An increase in any of the pesticide variables or farming intensity was associated with a higher proportion of declining species as expected.

Our analysis included both static and change-based variables. Because of the reduced temporal availability of pesticide data, we were limited to look for model fit based on variables measured at the midpoint of the bird trend period rather than estimating a change in these predictor variables over the period of interest. All of these static variables carry an implicit assumption that measurements made at the midpoint of the interval over which bird trends were calculated, are broadly representative of the entire period. We consider it meaningful that even with this handicap, the static variables ‘lethal pesticide risk ‘and ‘insecticide use’ emerged as more plausible than the pasture loss variables which we were able to measure over the entire period. Our results are all the more remarkable since any signal of acute pesticide effect in our analysis was undoubtedly ‘diluted’ by a gradual reduction in toxic insecticide use during the period of analysis [17].

Our results suggest that the use of lethally toxic insecticides cannot be ignored when trying to identify causes of grassland population declines in North America. Indeed, they offer a more plausible explanation for overall declines than does the oft-cited ‘habitat loss through agricultural intensification’. It was remarkable that loss of permanent pasture did not appear to be much of a predictor of grassland bird declines. The high correlations between our variables make it impossible to separate the direct effects of insecticides from the indirect ones and it is likely that both are operating. However, there is some indication that direct effects were most important during the period under consideration. Not only is ‘lethal risk’ a slightly better predictor than ‘insecticide use’ as judged by overall model weight but, herbicide use is a much weaker predictor. If the indirect effects of pesticides were the most important, we would expect herbicides to be contributing to grassland bird declines as noted elsewhere (e.g. [12]).

Many of the grassland species examined often have but a weak association with row or field crops where the bulk of insecticide use takes place. However, a number of factors need to be considered: 1) Mineau and colleagues showed that only a small proportion of total cropland need be treated with a dangerous pesticide to affect overall population trends [21]. Even a casual association with cropland may place birds at risk; 2) Although pastures are believed to receive much lower insecticide loads than other crops, alfalfa still carries the third highest lethal risk of any crop based on pesticide use [17]. Insecticides are used to control some rangeland pests such as grasshoppers; there are no reliable data on this but it is reasonable to assume that insecticide use on pasture may be proportional to insecticide use on field crops nearby; 3) The majority of species considered in this analysis (16/24) have been recorded killed in pesticide field trials despite the limited number of such studies (Table 1). Several of the grassland species showing the largest number of relative declines (e.g. horned lark, vesper sparrow) are species frequently picked up dead in the course of pesticide impact studies; 4) Pesticide drift is a well known phenomenon which may extend risk from a crop to an adjoining grassland or pasture.

In conclusion, it would be foolhardy for anyone to argue that habitat loss is of no importance to bird declines. However, we should be careful to consider pest control and specifically the use of highly toxic insecticides as a potential contributor to those declines. Unfortunately, information on pesticide use is often difficult to obtain or considered to be confidential, hampering any serious analysis of its true impact.
